# Macrophage‐Driven Bidirectional Exacerbation in Psoriasis‐Atherosclerosis Comorbidity: Insights From a Novel Mouse Model

**DOI:** 10.1155/mi/1377824

**Published:** 2026-07-26

**Authors:** Fangshun Tan, Yu Jiang, Ruizhi Wang, Ziquan Hu, Yuting Xia, Qi Pan, Liang Zhao, Jing Xu, Jiajia Lan, Menglu Liu, Jiafei Li, Mengyuan Wang, Wenjie Yan, Tianlang Zhao, Juan Tao, Weixian Yang

**Affiliations:** ^1^ Department of Cardiology, Fuwai Hospital, National Center for Cardiovascular Diseases of China, State Key Laboratory of Cardiovascular Disease, Chinese Academy of Medical Sciences and Peking Union Medical College, Beijing, China, cacms.ac.cn; ^2^ Cardiac Arrhythmia Center, Department of Cardiology, Fuwai Hospital, National Center for Cardiovascular Diseases, State Key Laboratory of Cardiovascular Disease, Chinese Academy of Medical Sciences and Peking Union Medical College, Beijing, China, cacms.ac.cn; ^3^ Department of Dermatology, Affiliated Union Hospital, Tongji Medical College, Huazhong University of Science and Technology, Wuhan, China, hust.edu.cn; ^4^ Department of Dermatology, Renmin Hospital of Wuhan University, Wuhan, China, rmhospital.com; ^5^ Beijing Key Laboratory of Xenotransplantation, Animal Experimental Centre, Fuwai Hospital, National Centre for Cardiovascular Disease, Chinese Academy of Medical Sciences and Peking Union Medical College, Beijing 100037, China, cacms.ac.cn; ^6^ Department of Geriatrics, Tongji Hospital, Tongji Medical College, Huazhong University of Science and Technology, 1095 Jiefang Avenue, Wuhan 430030, China, hust.edu.cn

**Keywords:** animal models, atherosclerosis, comorbidity, mouse, psoriasis

## Abstract

**Background:**

Psoriasis (PSO) and atherosclerosis (AS) are chronic inflammatory diseases that frequently coexist, with each condition exacerbating the other. However, the underlying mechanisms remain poorly understood, partly due to the lack of a suitable animal model that recapitulates this bidirectional comorbidity.

**Methods:**

PSO‐AS mouse models were constructed by combining high‐fat diet (HFD)–induced AS with imiquimod (IMQ)‐triggered psoriatic inflammation in ApoE^−/−^ mice. PSO severity was assessed via skin manifestation, hematoxylin and eosin (H&E) staining, PSO area and severity index (PASI) score, quantitative real‐time PCR (qRT‐PCR), and serum ELISA, while AS was assessed by en face Oil red O (ORO) staining of aorta, H&E staining, ORO staining, Sirius Red (SR) staining, and immunofluorescence (IF) staining of the aortic root section. Mechanistic insights were obtained via proteomics, machine learning (ML), and validation of macrophage polarization in both skin and plaques.

**Results:**

Chronic PSO model more closely resembles real‐world conditions than acute PSO model. Combining the IMQ application with a HFD, both the PSO‐AS group and the PSO group developed obvious psoriatic skin lesion, but the PSO‐AS group showed the highest epidermal thickness, PASI scores, and expression levels of psoriatic biomarkers (Interleukin‐17A [IL‐17A] and Interleukin‐23 [IL‐23]) and inflammatory factors (e.g., tumor necrosis factor alpha [TNF‐α] and Interleukin‐6 [IL‐6]), implying that AS may exacerbate PSO. Similarly, the PSO‐AS and AS groups both developed obvious plaque in the aorta, while the PSO‐AS group has the largest plaque area, necrotic core (NC) area, lipid content with the least fibrous cap (FC) thickness, and collagen content, illustrating that PSO conspicuously aggravated AS progression and led to an unstable plaque shift. Statistical analysis also confirms that PSO and AS mutually exacerbate each other, forming a vicious cycle that may be strongly associated with IL‐17. Proteomics and ML revealed marked M1 macrophage polarization in PSO‐AS mice. Validation by Western blot and IF showed upregulated M1 markers (CD86) and downregulated M2 markers (CD163) in both skin lesions and aortic roots, indicating that M1‐skewed macrophages fuel the vicious cycle. Besides, we explore that IFIT3 may serve as a promising biomarker for M1 macrophages in PSO‐AS skin.

**Conclusions:**

This novel PSO‐AS mouse model faithfully recapitulates the bidirectional exacerbation seen clinically and uncovers M1 macrophage polarization as a key driver of this comorbidity. The model provides a robust platform for mechanistic and therapeutic studies of PSO‐associated cardiovascular disease (CVD).

## 1. Introduction

Triggered by gene–environment interaction, psoriasis (PSO) is a common chronic papulosquamous skin disease affecting ~125 million people worldwide [1–3]. Substantial research indicates a significant correlation between severe PSO and cardiovascular disorders. PSO exhibits overlapping pathological features with atherosclerosis (AS) and other cardiovascular risk factors, suggesting shared disease mechanisms.

Severe PSO patients face doubled cardiovascular disease (CVD) risk, particularly for major adverse cardiovascular events (MACEs), myocardial infarctions (MIs), and stroke, confirmed by meta‐analysis and linked to disease severity/duration [4]. Studies in PSO patients have evaluated AS progression beyond clinical endpoints, revealing correlations with coronary artery calcification, elevated traditional risk factors, greater arterial stiffness, increased carotid intima‐media thickness, and an association linking femoral atherosclerotic plaque thickness to disease duration [5–8]. Wu and Poon’s study demonstrated a 74% reduction in MI risk among PSO patients treated with Tumor necrosis factor alpha (TNF‐α) inhibitors (hazard ratio [HR] 0.26, 95% confidence interval [CI] 0.12–0.56) compared to untreated controls [9]. However, biologic nonresponders showed minimal improvement in MACE, underscoring the therapy‐dependent nature of this cardioprotection [10, 11]. Supporting evidence from multiple cohort studies further suggests that biologics may reduce 50% MI incidence in PSO patients, reinforcing their potential role in mitigating cardiovascular risk beyond skin disease management [12, 13].

Currently, there is a lack of an animal model for PSO‐AS comorbidity. Such a model is needed to understand the molecular mechanisms behind the mutual exacerbation seen in clinical settings. The topical application of imiquimod (IMQ) for 5–7 days represents the most commonly adopted approach to establish psoriatic animal models. Though cost‐effective and operationally simple, this acute inflammatory model fails to fully recapitulate the chronic and relapsing nature of human PSO [14], not to mention combining this method with the AS model, which usually requires feeding ApoE^-/-^ mice with high‐fat diet (HFD) for more than 12 weeks [15].

We aimed to investigate the combined effects of prolonging the IMQ application time and reducing the dosage of IMQ in HFD‐fed ApoE^−/−^ mice. This study established a novel method for PSO‐AS animal model construction and recapitulated the mutual exacerbation phenomenon in PSO‐AS, with Interleukin‐17A (IL‐17A) acting as a key mediator and M1‐like macrophages driving tissue inflammation in both skin and vessels. Our model provided a simple and robust platform to uncover the biology and therapy for PSO‐AS.

## 2. Materials and Methods

### 2.1. Mouse Research

All animal experimental protocols used in this study were approved by the Experimental Animals Ethics Committee of Fuwai Hospital (Beijing; Approval Number 0108‐7‐500‐ZX(X)‐057), and all procedures met the National Institutes of Health guidelines. Male ApoE^−/−^ mice were purchased from Beijing Vital River Laboratory Animal Technology (Beijing, China) for all animal studies. All mice were maintained in the animal facilities under specific pathogen‐free conditions.

We initially assessed aortic damage in various PSO models. The most commonly used method for the PSO model, here referred to as acute PSO group (6–8 weeks, 18 mice per group), involved shaving the dorsal hair and then applying 62.5 mg of 5% IMQ cream (Aldara; 3M Pharmaceuticals) topically to the shaved area for 5–7 consecutive days. The chronic PSO group (6–8 weeks, 18 mice per group) innovatively involved dorsal hair removal from both the upper and lower back, followed by topical application of 50 mg of 5% IMQ cream to the shaved area. This treatment was repeated for five 5‐day cycles of IMQ with a 2‐day recovery period between cycles. The IMQ cream was applied to the upper back for the first 2 weeks and then to the lower back for the following 2 weeks. The normal group (6–8 weeks old, 18 mice per group) did not receive IMQ treatment, while all other conditions were kept consistent. Daily monitoring included measurements of body weight and assessments of the dorsal skin. At the end of the modeling stage, aortic tissues from three groups were collected for bulk RNA sequencing (RNA‐seq) analysis.

For the PSO‐AS group, male ApoE^−/−^ mice (6–8 weeks, 18 mice per group) were fed with HFD (research diets, D12108C, 40 kcal% fat, and 1.25% cholesterol) for 12 weeks until the endpoint. Based on Zhang et al.’s [16] previous exploration of modeling protocols for chronic PSO, we optimized and improved upon it to form this comorbidity modeling protocol. Given that early plaques typically form between 8 and 12 weeks, preliminary experiments were conducted at weeks 8, 11, and 14 during a HFD regimen [17–19]. The results indicated that week 8 was the optimal time point. After 8 weeks of HFD, the dorsal hair on the upper back of the mice was removed, in alliance with a topical application of 50 mg 5% IMQ cream on the shaved skin for five 5‐day cycles of IMQ with a 2‐day recovery period between IMQ cycles. Then, after 10 weeks of HFD, the dorsal hair on the lower back of the mice was removed, following the same IMQ cycle. The skin and weight were examined every day, and the dorsa were photographed. Finally, the mice were sacrificed after 12 weeks of HFD.

For the AS group, male ApoE^−/−^ mice (6–8 weeks, 18 mice per group) were fed with HFD for 12 weeks until the endpoint. After 8 weeks of HFD, the dorsal hair on the upper back of the mice was removed, and then, after 10 weeks of HFD, lower back hair was removed, with Vaseline application. Skin condition and body weight were recorded daily, with dorsal photographs taken for documentation. Following 12 weeks of HFD, the mice were euthanized for experiments.

For the PSO group, male ApoE^−/−^ mice (6–8 weeks, 18 mice per group) were fed with chow diet (CD) for 12 weeks until sacrifice. Following an 8‐week CD period, the mice underwent dorsal hair removal on the upper back, followed by topical treatment with 50 mg of 5% IMQ cream applied to the shaved area. This operation consisted of five 5‐day IMQ cycles, each separated by a 2‐day recovery interval. After a 10‐week CD period, the same IMQ protocol was repeated after shaving the lower dorsal hair for 2 weeks until euthanization. Daily monitoring included skin assessments, body weight measurements, and photographic documentation of the dorsal areas.

For the blank control (BC) group, male ApoE^−/−^ mice (6–8 weeks, 18 mice per group) were fed with CD for 12 weeks until sacrifice. Dorsal hair removal was performed on mice at specific intervals: upper back at week 8 and lower back at week 10 after CD, with Vaseline application. Daily assessments included skin condition, body weight measurements, and dorsal photography for documentation. After completing 12 weeks of CD, mice were euthanized for experimentation (Figure 1).

Figure 1Comparison of results from acute and chronic psoriasis modeling methods. *p*‐Values were shown as  ^∗^ *p*  < 0.05,  ^∗∗^ *p*  < 0.01, and  ^∗∗∗^ *p*  < 0.001. (A) Representative images of H&E staining (scale bar = 100 μm), showing epidermal hyperplasia and immune cell infiltration. (B) Summary bar graphs about fluctuations of epidermal thickness, weight, and PASI score. (C) Serum levels of IL‐6, TNF‐α, IL‐17A, and IL‐23 detected by ELISA. (D) The mRNA levels of IL‐17A, IL‐23, TNF‐α, S100a8, and S100a9 in skin lesions determined by qRT‐PCR (*n* = 6). (E) Identification of differentially expressed genes by limma, edgeR, and DESeq. (F) Volcano plot showed upregulated and downregulated DEGs, and Gene Ontology enrichment analysis revealed related molecular functions (chronic group vs. acute group). (G) Volcano plot showed upregulated and downregulated DEGs, and Gene Ontology enrichment analysis revealed related molecular functions (chronic group vs. normal group). (H) Volcano plot showed upregulated and downregulated DEGs, and Gene Ontology enrichment analysis revealed related molecular functions (acute group vs. normal group).

**Figure:**
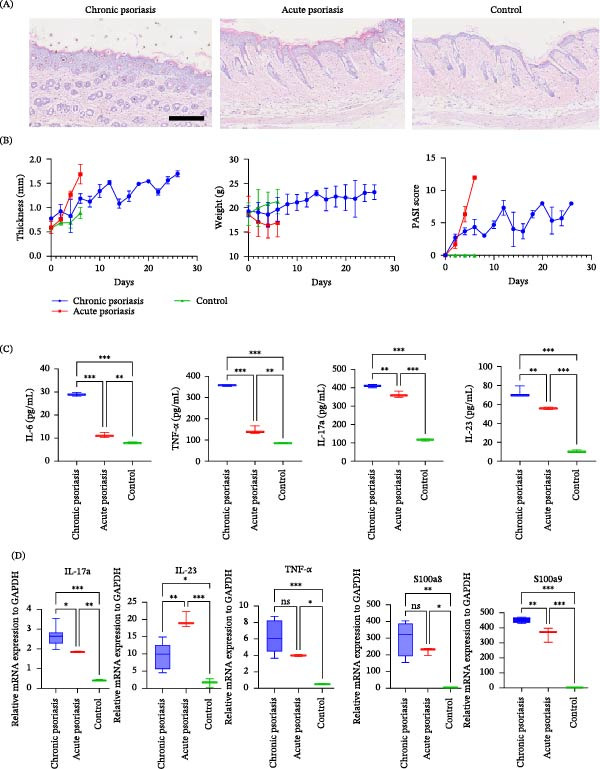

**Figure:**
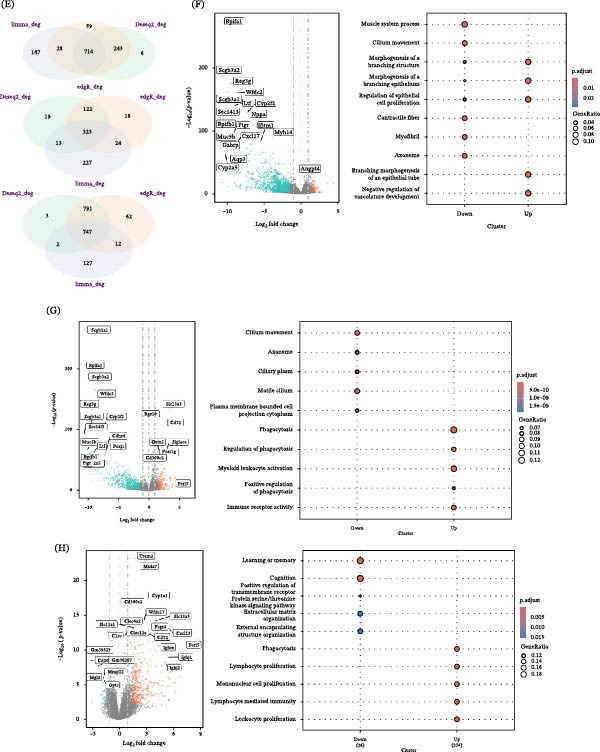

Sample size estimation was predicated on an initial experiment, with the primary outcomes assessed as epidermal thickness and plaque area. Preliminary experiments utilizing 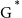
power software suggested a requisite sample size of 6 for each group to achieve an 80% power level and a 5% significant level (two‐sided). To account for tissue allocation across multiple downstream assays (histology, immunofluorescence (IF), quantitative real‐time PCR (qRT‐PCR), Western blot, ELISA, and proteomics), potential attrition from IMQ‐induced toxicity and HFD‐related complications, and sample size from previous articles, we used a total of 18 mice per group. The exact number of mice used for each specific assay is indicated in the corresponding figure legends.

After the acclimatization period, mice were randomly assigned to experimental groups (PSO‐AS, AS, PSO, and BC) using a computer‐generated random sequence (GraphPad QuickCalcs, CA, USA) with block randomization (block size of 4). Group allocation was concealed using opaque, sealed envelopes prepared by an independent researcher, which were opened sequentially at the time of cage assignment.

### 2.2. Measurement of Skin Scores

PSO severity was assessed via the PSO area and severity index (PASI) score. Over a 4‐week period, mouse skin thickness, erythema, and scaling were documented daily. Thickness underwent quantitative measurement using a micrometer, while erythema and scaling were rated on a 0–4 scale (0: no symptoms; 1: mild; 2: moderate; 3: severe; and 4: very severe). The PASI score was independently evaluated by three blinded researchers. Representative images of mouse skin of different sub‐PASI scores (0–4 for erythema, scaling, and thickness) and total PASI scores are provided in Supporting Information 1: Figure S1.

### 2.3. Spleen Index

Body weight of each mouse was monitored daily for the entire study period. At the endpoint, spleens were excised and gently rinsed with PBS to remove residual blood. Each spleen was weighed, and its mass together with the corresponding body weight was used to determine the organ index according to the following formulas: spleen index = spleen weight (mg)/mice weight (g).

### 2.4. Histology and IF Staining

Mouse back skin was fixed in 4% paraformaldehyde, embedded in paraffin, and cut into 5 μm sections. Hematoxylin and eosin (H&E) was performed to evaluate inflammatory infiltration and epidermal thickness. Hearts were dissected from the aortas, embedded in optimal cutting temperature compound (Leagene, China), and cut into 10‐µm‐thick cross sections for staining. Aortic roots’ histologic analysis was performed on frozen sections via H&E, Oil red O (ORO), and Sirius Red (SR) staining according to the manufacturer’s instructions (Solarbio, China). The sections were then observed by optical microscopy (Olympus, Japan). The back‐skin sections of mice from each group were deparaffinized and rehydrated, followed by antigen retrieval. For IF analysis, the sections were incubated with antibodies against CD68 (Proteintech, 28058‐1‐AP), α‐SMA (Proteintech, 67735‐1‐Ig), CD86 (Proteintech, 30691‐1‐AP), CD163 (Proteintech, 68218‐1‐Ig), F4/80 (Abcam, ab320060), and IFIT3 (Proteintech, 15201‐1‐AP). They were then detected by fluorescently labeled secondary antibodies, and the nucleus was stained with DAPI (Sigma‐Aldrich, F6057). The sections of IF staining were observed under the Leica SP8 confocal microscopy. The plaque size was quantified as the proportion of the lesion area/lumen area. The relative necrotic core (NC) area was calculated by NC area/plaque area. Lipid content was assessed by ORO staining. Collagen content was assessed by SR staining. Macrophage and smooth muscle cell content were assessed by IF staining. Plaque composition was quantified as a percentage of total lesion area of each component. Vulnerability plaque index (VPI) was calculated as VPI = (% NC area + % CD68 area)/(% SMA area + % collagen area) according to Silvestre‐Roig’s study [20]. For en face staining, the entire aorta (from the aortic root to the iliac bifurcation) was meticulously dissected to remove perivascular adipose tissue and longitudinally incised to expose the luminal surface. The prepared aortas were pinned en face and stained with ORO (Sigma‐Aldrich, 00625) to visualize atherosclerotic plaque deposits. Subsequently, the specimens were imaged under an optical microscope, and ORO‐positive lesion areas were quantified. Data analysis was performed by investigators blind to the mouse group via ImageJ software. Quantification of the plaque area, NC area, lipid content, collagen content, and IF signals was performed by investigators blinded to group allocation. Prior to performing quantification of IF images, the images were blinded and randomized using a custom ImageJ macro.

### 2.5. qRT‐PCR

Total RNA was extracted from mouse skin samples using TRIzol reagent (Invitrogen, CA, USA). Briefly, homogenized skin samples were mixed with chloroform and centrifuged at 12,000 g for 30 min at 4°C, and the aqueous phase was collected. RNA was precipitated with isopropanol, washed with 75% ethanol, and dissolved in RNase‐free water. RNA concentration and purity were assessed using a NanoDrop 2000 spectrophotometer (Thermo Fisher Scientific, Massachusetts, USA). Then, 1000 ng of total RNA per sample was reverse transcribed into cDNA (Roche, Shanghai, China) in a total reaction volume of 20 µL. The reverse transcription reaction was performed under the following conditions: 10 min at 25°C, followed by 30 min at 55°C, then 5 min at 85°C, and held at 4°C. Then, real‐time PCR was performed using SYBR Green Master Mix (Thermo Fisher Scientific, Massachusetts, USA). The reaction mixture (12 µL) contained 6 µL of SYBR Green Master Mix, 0.5 µL each of forward and reverse primers (10 µM), and 5 µL of diluted cDNA (1:50 dilution). The thermal cycling protocol was as follows: initial denaturation at 95°C for 2 min, followed by 40 cycles of 95°C for 15 s, annealing at 60°C for 30 s, and extension at 72°C for 30 s. A melt curve analysis was performed from 60 to 95°C to verify amplification specificity. Skin samples from six mice per group were used as biological replicates, and each sample was run in triplicate. The mRNA levels of the target genes were normalized relative to those of GAPDH using the following formula: relative mRNA expression = 2 ^−(△Ct of target gene−△Ct of GAPDH)^, where Ct is the threshold cycle value. The primers are listed in Table 1.

**Table 1:** Sequences of primers used for amplification of genes.

Gene	Primer nucleotide sequence
IL‐17A	Forward: 5’‐CCTCACACGAGGCACAAGTG‐3’
	Reverse: 5’‐CTCTCCCTGGACTCATGTTTGC‐3’
IL‐17F	Forward: 5’‐TGCTACTGTTGATGTTGGGAC‐3’
	Reverse: 5’‐AATGCCCTGGTTTTGGTTGAA‐3’
IL‐23	Forward: 5’‐CAGCAGCTCTCTCGGAATCTC‐3’
	Reverse: 5’‐TGGATACGGGGCACATTATTTTT‐3’
IL‐6	Forward: 5’‐ TAGTCCTTCCTACCCCAATTTCC‐3’
	Reverse: 5’‐ TTGGTCCTTAGCCACTCCTTC‐3’
IFN‐γ	Forward: 5’‐ AAGACTGTGATTGCGGGGTT‐3’
	Reverse: 5’‐ ATCTGAGTTCAGTCAGCCGC‐3’
S100a8	Forward: 5’‐GTCCTCAGTTTGTGCAGAATATAAA‐3’
	Reverse: 5’‐GCCAGAAGCTCTGCTACTCC‐3’
S100a9	Forward: 5’‐TGGCAACCTTTATGAAGAAAGAGA‐3’
	Reverse: 5’‐GTGGGTTGTTCTCATGCAGC‐3’
CXCL15	Forward: 5’‐TGTTCACAGGTGACTGCTCC‐3’
	Reverse: 5’‐AGCCCATAGTGGAGTGGGAT‐3’
TNF‐α	Forward: 5’‐ATCCGCGACGTGGAACTG‐3’
	Reverse: 5’‐ ACCGCCTGGAGTTCTGGAA‐3’
GAPDH	Forward: 5’‐GCTGAGTATGTCGTGGAGT‐3’
	Reverse: 5’‐GTTCACACCCATCACAAAC‐3’

### 2.6. ELISA

For the detection of psoriatic biomarkers and proinflammatory cytokine in serum, ELISA kits (Mouse IL‐17A ELISA kit: Elabscience, E‐EL‐M0047; Mouse Interleukin‐23 [lL‐23] ELISA kit: Elabscience, E‐EL‐M0731; high sensitivity mouse TNF‐α ELISA kit: Elabscience, E‐HSEL‐M0009; and high sensitivity mouse Interleukin‐6 [lL‐6] ELISA kit: Elabscience, E‐HSEL‐M0003) were used in accordance with the manufacturer’s instructions. Mouse blood samples were collected individually from each mouse at the endpoint under deep anesthesia with pentobarbital via cardiac puncture. Mouse blood samples from the PSO‐AS, AS, PSO, and BC groups were centrifuged at 1000 g for 10 min at 4°C to obtain the serum.

### 2.7. Western Blot

Western blot was employed to analyze proteins related to macrophage polarization. Skin tissue was prepared using RIPA (Thermo Fisher Scientific, 89900) with protease and phosphatase inhibitors. The protein samples were separated by SDS‐PAGE and transferred to PVDF membranes. After blocking for 30 min using QuickBlock Western blocking buffer at room temperature, membranes were incubated with primary antibodies against CD86 (Proteintech, 30691‐1‐AP), IFIT3 (Proteintech, 15201‐1‐AP), and GAPDH (Proteintech, 60004‐1‐Ig) at 4°C overnight. Then, membranes were washed by TBST three times and incubated with secondary antibodies for 40 min at room temperature, visualized by HRP substrate peroxide solution and HRP substrate luminol solution (EMD Millipore Corporation, Burlington, USA). The immunoblot band intensity was quantified with ImageJ software (NIH, MD, USA). ImageJ analysis was performed by an investigator blinded to group allocation. The analyst was unaware of the sample identities until all quantitative measurements were completed.

### 2.8. RNA‐Seq, Proteomic Sequencing, and Bioinformatic Analysis

For transcriptome analysis, total RNA was extracted from mouse aorta tissues. mRNA was enriched using Oligo(dT) magnetic beads, fragmented, and reverse‐transcribed into double‐stranded cDNA. After end repair, A‐tailing, adapter ligation, and PCR amplification, libraries (insert size 250–300 bp) were sequenced on an Illumina NovaSeq 6000 platform (PE150). Raw reads were filtered with fastp, and clean reads were aligned to the mouse reference genome GRCm39 using HISAT2. Transcripts were assembled and quantified by StringTie, and gene expression levels were calculated as FPKM. Differential expression analysis was performed with limma, DESeq2, and edgeR, thus selecting intersected common differentially expressed genes (DEGs) for further analysis.

For proteomic sequencing, skin tissue proteins were extracted and digested with trypsin, and the resulting peptides were subjected to data‐independent acquisition (DIA) mass spectrometry (MS). Peptide separation was performed by liquid chromatography, followed by tandem MS (LC‐MS/MS). Database searching was carried out with DIA‐NN (FDR < 1%), and protein quantification was based on peptide‐spectrum matches (PSMs). Differential proteins between groups were identified using “DEP” package of R software, with a *p*  < 0.05 and |log fold change| > 1.

Gene Ontology (GO), Kyoto Encyclopedia of Genes and Genomes (KEGG), and gene set enrichment analysis (GSEA) were performed for both DEGs or differentially abundant proteins to identify biological processes and signaling pathways. Six machine learning (ML) methods, namely, least absolute shrinkage and selection operator (LASSO), support vector machine (SVM) algorithm, decision tree (DT) algorithm, random forest (RF) algorithm, XGBoost, and Boruta algorithm, were used to identify the core differential proteins in proteomics.

### 2.9. Statistical Analysis

All data were analyzed using GraphPad Prism software (version 9.0) and R 4.4.1. Data are presented as mean ± SD. Prior to parametric analyses, the normality of data distribution was assessed using the Shapiro–Wilk test, and homogeneity of variances was evaluated using Levene’s test (or Brown–Forsythe test for Welch’s ANOVA). For comparisons between two groups, a two‐tailed unpaired Student’s *t*‐test was used when normality and variance homogeneity assumptions were met; otherwise, the Mann–Whitney *U* test was applied. For comparisons involving more than two groups under a single factor, one‐way ANOVA was performed. When variances were equal, Tukey’s multiple comparison test was used as the post hoc test; when variances were unequal (by Brown–Forsythe test), Welch’s ANOVA followed by Dunnett’s T3 test was applied. For experiments with two independent variables, two‐way ANOVA was used to evaluate the main effects and their interaction. When a significant interaction or main effect was detected, Tukey’s honestly significant difference (HSD) test was applied for multiple pairwise comparisons. For data that did not pass the normality test even after transformation, the Kruskal–Wallis test followed by Dunn’s post hoc test was used. Correlation analyses were performed using Spearman’s rank correlation coefficient. Mediation analysis was conducted using the “mediation” R package. The number of replicates per experiment is indicated in the corresponding figure legends. A two‐sided *p*‐value < 0.05 was considered statistically significant.  ^∗^ *p*  < 0.05,  ^∗∗^ *p*  < 0.01, and  ^∗∗∗^ *p*  < 0.001.

## 3. Results

### 3.1. Chronic PSO Exacerbates Inflammation and Aortic Damage

As mentioned above, we collected aorta samples from three groups, i.e., chronic PSO, acute PSO, and control group at the end of the process and performed bulk RNA‐seq sequencing, illustrating whether chronic PSO could lead to aortic damage and applied into comorbidity model.

After the IMQ challenge, both acute and chronic PSO groups exhibited skin manifestations, including thickening and erythema. However, the chronic PSO group exhibited more pronounced skin changes. Histological examination using H&E staining revealed classic features in the chronic group, such as epidermal thickening, hyperkeratosis, and parakeratosis (Figure 1A). Throughout the modeling period, both acute and chronic groups showed significant increases in PASI scores and skin thickness induced by IMQ application. Notably, the chronic group maintained a relatively stable body weight, while the acute group experienced a sharp decline (Figure 1B). Systemic inflammation was compared across the three groups as well. In peripheral blood serum samples, levels of inflammatory mediators—such as IL‐17A, IL‐23, TNF‐α, and IL‐6—were elevated markedly in the chronic PSO model group (Figure 1C). Similarly, mRNA expression of cytokines (IL‐17A and TNF‐α) and inflammatory markers (S100a8 and S100a9) was significantly higher in the chronic group compared to the acute or control groups (Figure 1D).

Subsequent analysis of aortic transcriptomic data was performed across three comparisons: chronic vs. acute, chronic vs. normal, and acute vs. normal. DEGs were identified using R packages—limma, edgeR, and DESeq2—and consensus DEGs from all three methods were selected for further investigation (Figure 1E). In the chronic vs. acute comparison, the volcano plot showed 69 upregulated and 678 downregulated DEGs. GO enrichment analysis revealed that these DEGs were significantly associated with terms like “branching morphogenesis of an epithelial tube,” “negative regulation of vasculature development,” “morphogenesis of a branching structure,” “morphogenesis of a branching epithelium,” and “regulation of epithelial cell proliferation.” These findings suggested that the chronic PSO model may have more detrimental effects on vascular morphology and function than the acute PSO model (Figure 1F).

In the chronic vs. normal comparison, 173 upregulated and 541 downregulated DEGs were identified. GO analysis of upregulated DEGs revealed enrichment in biological processes, including “phagocytosis,” “regulation of phagocytosis,” “myeloid leukocyte activation,” “positive regulation of phagocytosis,” and “immune receptor activity,” indicating enhanced immune activation and phagocytic function (Figure 1G). In the acute vs. normal comparison, 290 upregulated and 33 downregulated DEGs were found. GO analysis revealed that upregulated DEGs were enriched in terms like “phagocytosis,” “lymphocyte proliferation,” “mononuclear cell proliferation,” “lymphocyte‐mediated immunity,” and “leukocyte proliferation,” suggesting similar immune activation (Figure 1H). In summary, although both acute and chronic PSO models induced immune activation, the chronic model exhibited enhanced immune response and phagocytic activity. The chronic PSO model leads to extensive and severe vascular injury compared to the acute model, which is likely due to prolonged immune activation and persistent inflammatory responses.

### 3.2. AS Exacerbates Psoriatic Skin Inflammation

**Figure 2:**
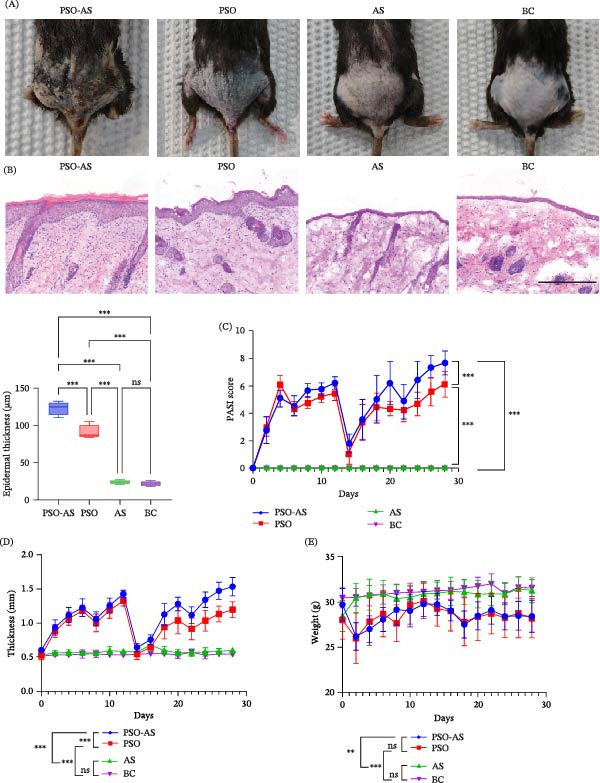
AS promoted skin inflammation in IMQ‐induced mouse model. *p*‐Values were shown as  ^∗^ *p*  < 0.05,  ^∗∗^ *p*  < 0.01, and  ^∗∗∗^ *p*  < 0.001. (A) Representative clinical photographs. (B) Representative images of H&E staining and summary bar graphs of epidermal thickness (scale bar = 100 μm), showing epidermal hyperplasia and immune cell infiltration (*n* = 6). (C) Changes in clinical severity, measured by PASI score. (D) Skin thickness measured by micrometer. (E) Body weight changes in all groups during the experimental period.

The grouping and model construction procedures have been clarified in detail in Section 2 (Supporting Information 2: Figure S2). IMQ‐induced mouse dermatitis well‐mimics human PSO [21]. We found that both the PSO‐AS and PSO groups developed severe dermatitis according to clinical signs of PSO, such as skin thickening, erythema, and scaling, and histologic analysis exhibited obvious classic keratinocyte hyperproliferation, hyperkeratosis, parakeratosis, and Munro microabscesses after chronic IMQ challenge (Figure 2A,B). The epidermal thickness in the PSO‐AS group was apparently larger than the PSO group, with the AS and BC groups displaying virtually no dermatitis (Figure 2B). During the whole period, the PSO‐AS group owned the highest PASI score and largest skin thickness (Figure 2C,D), even compared with the PSO group, implying that AS exacerbated psoriatic skin inflammation. Of note, despite receiving HFD, the PSO‐AS group maintained the lowest body weight, matching that of the PSO group at the endpoint (Figure 2E).

### 3.3. Systemic Inflammation and Immune Dysregulation in Comorbidity

**Figure 3:**
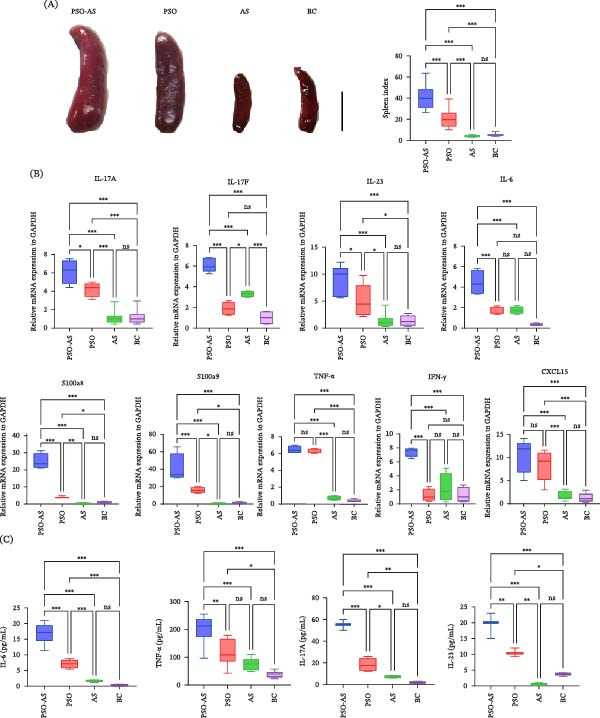
Systemic inflammation and immune profiling in mouse models. *p*‐Values were shown as  ^∗^ *p*  < 0.05,  ^∗∗^ *p*  < 0.01, and  ^∗∗∗^ *p*  < 0.001. (A) Representative photographs of the spleen (left) and spleen index (right) of mice from each group. Scale bar = 10 mm. *n* = 8. (B) The mRNA levels of IL‐17A, IL‐17F, IL‐23, IL‐6, S100a8, S100a9, TNF‐α, IFN‐γ, and CXCL15 in skin lesions determined by qRT‐PCR (*n* = 6). (C) Serum levels of IL‐6, TNF‐α, IL‐17A, and IL‐23 detected by ELISA (*n* = 6).

During the 12‐week experimental period, survival rates were 100% (18/18) in the BC, PSO, and AS groups and 94.4% (17/18) in the PSO‐AS group. One mouse in the PSO‐AS group died unexpectedly during the second hair removal procedure under anesthesia at week 10. The increase in spleen weight, given that the spleen is the largest immune organ, suggests a significant boost in the number of immune cells [22]. At the endpoint, we observed significant spleen enlargement after IMQ treatment. The PSO‐AS group had the highest spleen index, reflecting the most intense immune inflammatory response in this group, suggesting that AS may aggravate PSO through systemic inflammation (Figure 3A). Compared to the AS or BC group, the mRNA levels of cytokines (IL‐17A, IL‐17F, IL‐23, IL‐6, TNF‐α, and interferon‐γ [IFN‐γ]), antimicrobial peptides (S100a8 and S100a9), and chemokines (CXCL15) in mouse skin were conspicuously elevated in the PSO‐AS and PSO groups (Figure 3B). To be mentioned, the PSO‐AS group exhibited the highest S100a8, S100a9, IL‐17A, IL‐17F, IL‐6, and IL‐23 expression, illustrating the PSO‐AS group with the most intense skin inflammation and most severe psoriatic condition. In the peripheral blood serum samples, serum inflammatory mediators, such as IL‐17A, IL‐23, TNF‐α, and IL‐6, were elevated most in PSO‐AS mice (Figure 3C), hinting at Th17‐driven inflammation.

### 3.4. PSO Accelerates Atherosclerotic Plaque Progression and Vulnerability

**Figure 4:**
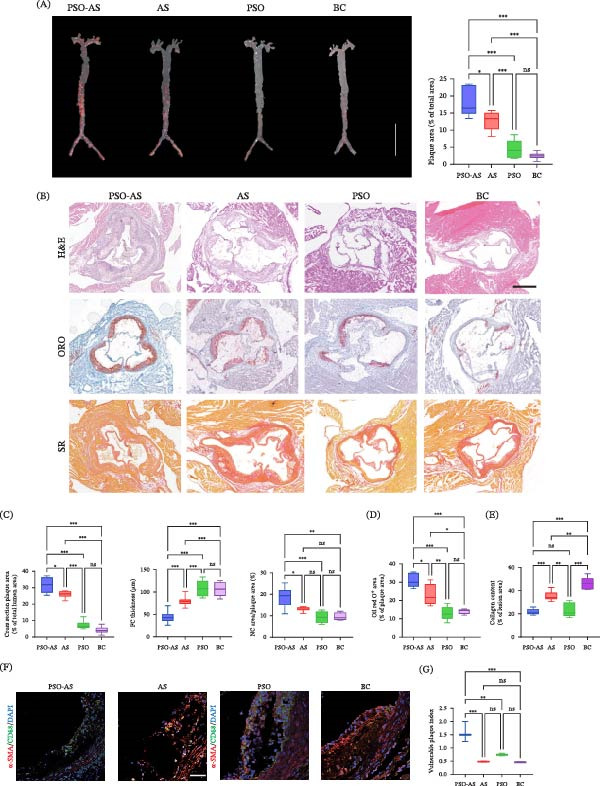
PSO aggravated AS, insights from plaque characterization and vulnerability assessment. *p*‐Values were shown as  ^∗^ *p*  < 0.05,  ^∗∗^ *p*  < 0.01, and  ^∗∗∗^ *p*  < 0.001. (A) Representative images of en face ORO‐stained total aortas (left) and quantification of plaque area (right). Scale bar = 10 mm. *n* = 6. (B) Pathological detections on cross sections from aortic roots by H&E (top), ORO (middle), and SR (bottom) in different groups. Scale bar = 500 μm. Quantitative analysis on (C) plaque area, FC thickness, and relative NC area; (D) lipid content; and (E) collagen content. *n* = 6. (F) Representative immunofluorescent images displaying overlapping α‐SMA (red) and CD68 (green) staining. Scale bar = 50 μm. (G) Plaque vulnerability measured by VPI index. *n* = 6.

Psoriatic inflammation dramatically exacerbated AS in the PSO‐AS comorbidity model, driving plaque progression and destabilization: Quantitative analysis revealed a 122% larger aortic plaque area in the entire arterial en face ORO staining (*p*  < 0.05) (Figure 4A) and 1.4‐fold increase in cross‐sectional lesions at aortic roots (*p*  < 0.05) (Figure 4B, top and Figure 4C, left), compared with AS group. Critically, compared with AS mice, plaques in PSO‐AS mice exhibited high‐risk features: fibrous cap (FC) thinned by 44% (*p*  < 0.001) (Figure 4B, top and Figure 4C, middle), NC expanded 1.44‐fold (*p*  < 0.05) (Figure 4B, top and Figure 4C, right), lipid content expanded 1.34‐fold (*p*  < 0.05) (Figure 4B, middle and Figure 4D), and collagen dropped 39% (*p*  < 0.001) (Figure 4B, bottom and Figure 4E). Cellular shifts confirmed vulnerability—macrophage infiltration (CD68^+^) surged while smooth muscle cells (α‐SMA^+^) decreased; these consequently composite VPI in PSO‐AS—224% higher than AS (*p*  < 0.001) (Figure 4F,G)—confirming rupture‐prone phenotypes driven by PSO‐associated systemic inflammation [20].

### 3.5. Synergistic Effects and IL‐17A Mediation in PSO‐AS Comorbidity

**Figure 5:**
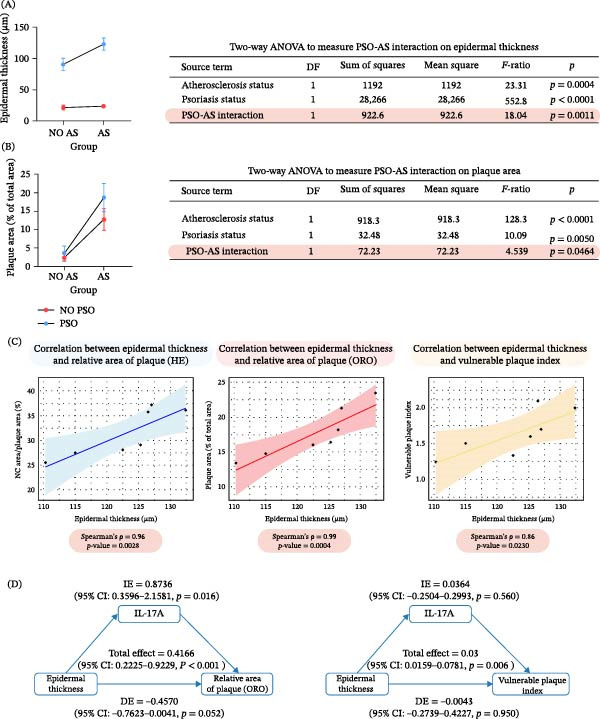
Skin inflammation and atherosclerotic plaque have correlations and exhibit a synergistic exacerbation. (A) Two‐way ANOVA analysis shows that PSO and AS had biological synergistic exacerbation on epidermal thickness in the PSO‐AS group. (B) Two‐way ANOVA analysis shows that PSO and AS had biological synergistic exacerbation on plaque area in the PSO‐AS group. (C) Correlations between epidermal thickness and the situation of plaque (HE‐stained plaque area, ORO‐stained plaque area, and vulnerable plaque index). (D) Mediation analysis to explore whether IL‐17A mediates the relationship between skin inflammation and atherosclerotic plaque.

The results from the previous analysis show that the PSO‐AS group exhibited the highest levels of skin inflammation and atherosclerotic plaque markers. However, this alone does not conclusively demonstrate a synergistic effect (“1 + 1 > 2”) between the two factors. We employed two‐way ANOVA to investigate whether PSO and AS exhibit biological synergistic exacerbation, using chronic PSO induction and HFD feeding as two factors. Epidermal thickness and aortic plaque ORO staining were used as indicators to measure the severity of PSO and AS, respectively. The results indicated that PSO and AS exhibit a synergistic exacerbation (*p* for PSO‐AS interaction < 0.05) (Figure 5A,B). We established a comorbidity model where the two conditions mutually exacerbate each other, rather than merely superimposing two independent models. Using the data of the PSO‐AS group, we then conducted an exploratory correlation analysis between skin inflammation severity (epidermal thickness) and plaque situation (plaque area and vulnerability). The results showed a significant positive correlation between the severity of skin inflammation and plaque severity (*p*  < 0.05) (Figure 5C). We also conducted a mediation analysis to explore whether IL‐17A mediates the relationship between skin inflammation and atherosclerotic plaque. The results confirmed that IL‐17A mediates the effect between epidermal thickness and plaque area (*p* for IE < 0.05) (Figure 5D).

### 3.6. M1 Macrophage Polarization Fuels Bidirectional Exacerbation in PSO‐AS Comorbidity

**Figure 6:**
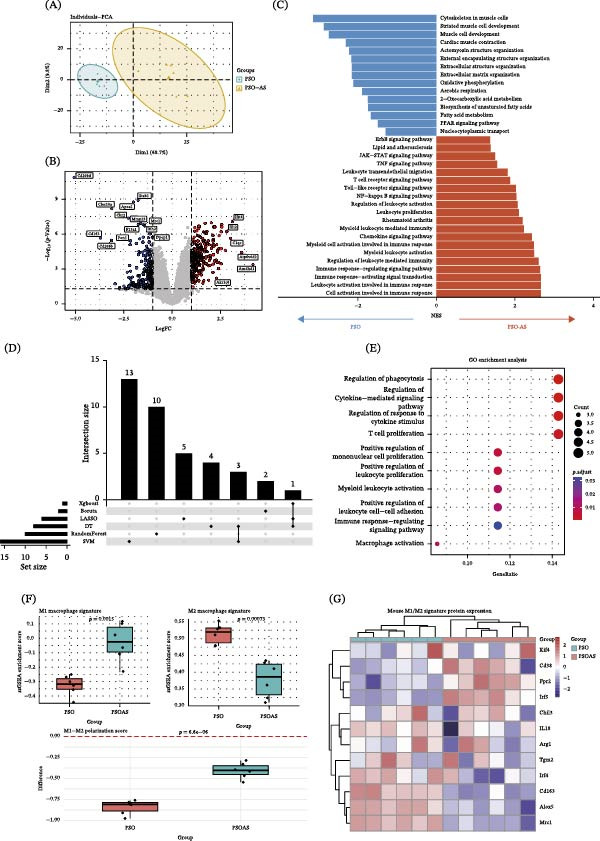
Proteomic profiling and machine learning reveal M1 macrophage polarization as a key signature in PSO‐AS comorbidity. (A) PCA plot of skin proteomics from PSO‐AS and PSO groups. (B) Volcano plot of differentially expressed proteins. Red and blue dots represent significantly upregulated and downregulated proteins, respectively. (C) GSEA plot for highlighted enriched pathways. (D) Upset plot depicted the core selected proteins by six ML methods. (E) GO enrichment analysis of ML‐selected proteins. (F) M1 and M2 gene set scoring in skin tissues of PSO‐AS and PSO groups. Up: left, ssGSEA enrichment score for the M1 macrophage gene signature; right, ssGSEA enrichment score for the M2 macrophage gene signature. Down: M1–M2 polarization score, calculated as the difference between M1 and M2 ssGSEA scores. (G) Heatmap of M1/M2 signature protein expression profiles.

We next investigated the potential mechanism of bidirectional exacerbation in PSO‐AS comorbidity. Skin tissue from the PSO‐AS and PSO groups was harvested for proteomic sequencing, exploring why skin lesions in the PSO‐AS group were much serious than the PSO group. Principal component analysis (PCA) showed that samples from the PSO‐AS and PSO groups distinguish from each other apparently (Figure 6A). Based on the threshold of *p*‐value < 0.05 and |log fold change| > 1, 255 upregulated proteins and 190 downregulated proteins were identified (Figure 6B). GSEA analysis revealed a distinctive pattern in PSO‐AS, such as “Lipid and atherosclerosis,” “TNF signaling pathway,” “Leukocyte transendothelial migration,” “regulation of leukocyte activation,” “leukocyte proliferation,” and “myeloid leukocyte mediated immunity,” hinting at the magnified inflammation, imbalanced immunity, and myeloid leukocyte activation (Figure 6C). Next, we employed LASSO, SVM, DT, RF, XGBoost, and Boruta algorithms to find the core differentially expressed proteins (Figure 6D). Proteins identified by each algorithm are summarized in Table 2, with the detailed selection process shown in Supporting Information 3: Figure S3. GO enrichment analysis of ML‐selected proteins further illustrated the unique role of macrophages in PSO‐AS comorbidity, with highlighted terms “regulation of phagocytosis,” “positive regulation of leukocyte proliferation,” “myeloid leukocyte activation,” and “macrophage activation” (Figure 6E). M1 and M2 gene set scoring revealed significantly higher M1 scores in the PSO‐AS group compared to the PSO group [23], indicating macrophage‐driven inflammation in exacerbated skin lesions (Figure 6F,G).

**Table 2:** Core differentially expressed proteins identified by different ML methods.

ML methods	Proteins
LASSO	Cd209d, Slc11a1, Apoa1, Stab1, Isg15, Hmgcs2
SVM	Fgd3, Stap1, Hsd11b1, Cyp2c29, Clec10a, Cd163, Itgb7, Cd209b, Cand2, Zbp1, Clec4a, Abca9, Col3a1, Siglec12, Mthfr, Arhgap30
RF	F13a1, Igf2bp2, Plin4, Ptpn22, Abat, Syk, C2cd6, Rnf213, Lcp2, Slamf7
Boruta	Lrrc58, Ifit1
DT	Atp6v0d2, Cd163, Cd209b, Cd209d, Ifit3, Amdhd1, Clec10a, C1qc
XGBoost	Cd209d

**Figure 7:**
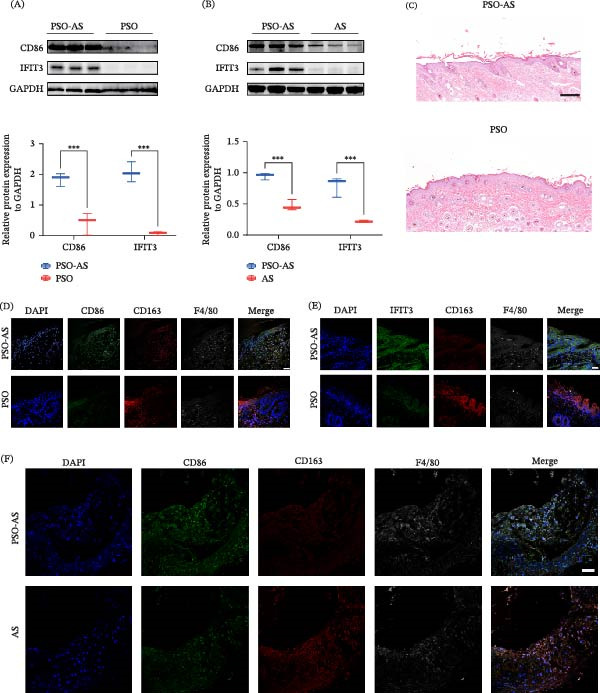
M1 macrophage polarization validation in both skin lesions and aortic roots.  ^∗^ *p*  < 0.05;  ^∗∗^ *p*  < 0.01; and  ^∗∗∗^ *p*  < 0.001. (A) Western blot analysis reveals elevated M1 marker, CD86, and IFIT3 expression in PSO‐AS mouse skin, compared to PSO mouse (*n* = 3). (B) Western blot analysis reveals elevated M1 marker, CD86, and IFIT3 expression in PSO‐AS mouse aorta, compared to AS mouse (*n* = 3). (C) Representative images of H&E staining of PSO‐AS and PSO mouse skin. Scale bar = 200 μm. (D) IF staining of CD86, CD163, and F4/80 in the dorsal skin from PSO‐AS and PSO mice. Scale bar = 50 μm. (E) IF staining of IFIT3, CD163, and F4/80 in the dorsal skin from PSO‐AS and PSO mice. Scale bar = 50 μm. (F) IF staining of CD86, CD163, and F4/80 in the aortic roots from PSO‐AS and AS mice. Scale bar = 50 μm.

To validate the above findings, we next examined the macrophage polarization in PSO‐AS comorbidity. As shown in Figure 7A, the M1 marker, CD86, was apparently overexpressed in PSO‐AS mouse skin, compared with the PSO group. According to Wu et al. [24], IFIT3 is a promising biomarker of M1 macrophage polarization in PSO, as we also detected IFIT3 by ML methods, we examined IFIT3 level in PSO‐AS mouse skin. Surprisingly, IFIT3 is upregulated in PSO‐AS mouse skin, while no obvious band was observed in the PSO group, hinting that IFIT3 might be a high‐specificity biomarker for M1 polarization in PSO‐AS comorbidity (Figure 7A). To determine whether psoriatic inflammation also drives M1 polarization in the vascular wall, we collected aortas from the PSO‐AS and AS groups. As shown in Figure 7B, CD86 expression was significantly higher in the PSO‐AS aorta, suggesting the enhanced M1 polarization in the PSO‐AS vessel. Similarly, IFIT3 is obviously upregulated in the aortas from the PSO‐AS group, while no obvious band was found in the AS group (Figure 7B). Thus, IFIT3 might be specifically induced by PSO‐driven inflammation in the atherosclerotic vessel wall. H&E staining displayed the basic structure of the skin in these two groups (Figure 7C). We next conduct IF experiments in the skin sections of the PSO‐AS group and PSO group mice. Compared with the PSO group, the PSO‐AS group showed stronger CD86 (M1) and weaker CD163 (M2) fluorescence, along with increased F4/80 fluorescence (Figure 7D). These findings reinforce M1 polarization and enhanced macrophage infiltration. Additionally, a similar pattern was observed, with IFIT3 upregulated in PSO‐AS skin lesions, further suggesting the potential of IFIT3 as M1 polarization marker in PSO‐AS comorbidity (Figure 7E).

As bidirectional exacerbation was confirmed in both skin and aorta plaques, we then examined macrophage polarization in aortic roots. IF staining results showed that, compared with the AS group, IMQ treatment significantly upregulated CD86 and F4/80 levels and downregulated CD163 levels in PSO‐AS groups (Figure 7F). These results indicated that macrophage infiltration and M1 to M2 shift exist in both exacerbated aorta and skin lesions, which may be a common mechanism in PSO‐AS comorbidity.

## 4. Discussion

This study establishes a novel murine model of PSO‐AS comorbidity that accurately replicates the bidirectional exacerbation observed in patients. By integrating prolonged, cyclic IMQ application with chronic HFD feeding in ApoE^−/−^ mice, this model overcomes the limitations of acute inflammation models and reveals the synergistic crosstalk between cutaneous and vascular inflammation, especially M1 macrophage polarization.

The most common method in PSO research involves the local application of 62.5 mg of IMQ for 5–7 consecutive days [25], due to its simplicity, feasibility, and ability to closely replicate clinical outcomes. However, this method is unsuitable for studying other PSO‐related diseases, as it focuses on acute inflammation. A chronic PSO model is necessary to better simulate the relationship between PSO and AS. Thus, we set the PSO modeling period to 4 weeks, dividing the mouse’s back into two regions. The drug was applied sequentially to each region to establish a chronic model [16]. Several studies attempted to model PSO‐AS comorbidity, each with distinct strengths and limitations. Xie et al. [26] applied 62.5 mg of IMQ for 5 days to ApoE^-^/^-^ mice, with normal diet kept. They observed PSO‐like skin lesions and elevated serum total cholesterol (TC) and low‐density lipoprotein (LDL) but did not assess atherosclerotic plaques [26]. Although lipid metabolism disturbances were observed, the major limitation is the 5‐day IMQ application, which cannot mimic the chronic relapsing nature of human PSO, along with the absence of vascular pathology evidence. Subsequently, Madsen et al. [27] tried a similar short‐term IMQ application in LDLR^-^/^-^ mice and found no acceleration of AS, suggesting that brief skin inflammation is insufficient to drive plaque formation. Baumer et al. [28] generated K14‐Rac1V12^–/+^ transgenic mice with keratinocyte‐specific constitutive Rac1 activation, which spontaneously develop chronic psoriatic dermatitis. When crossed with Srb1^-^/^-^ApoER61^H/H^ hyperlipidemic mice, they observed increased aortic plaque formation and identified macrophage SOD2 dysfunction and cholesterol crystal formation. While this model captures chronic skin inflammation, the strong gain‐of‐function mutation does not represent the complex gene‐environment interactions in most PSO patients. Moreover, the triple‐transgenic breeding is laborious and limits widespread use. Huang et al. [29] used a daily topical application of IMQ on the ears for 14 days, combined with HFD in ApoE^-^/^-^ mice. The findings demonstrate that psoriatic inflammation drives IL‐17 production from CD4^+^ T cells, which promotes collagen deposition and crosslinking in both skin and artery walls. This remodeling reduces interstitial space, physically trapping lipoproteins such as HDL and LDL and thereby impeding their transit from tissues to plasma. Consequently, IL‐17 contributes to accelerated AS and increased vascular stiffness. Importantly, neutralizing IL‐17 or inhibiting lysyl oxidase effectively reverses these pathological changes, highlighting potential therapeutic targets for cardiovascular comorbidity in PSO [29]. However, the IMQ treatment was only 14 days, not a chronic cycle, and the study focused on lipoprotein entrapment rather than on immune cell polarization, particularly macrophages. Baral et al. [30] focused on the Th9/IL‐9 axis using three models: IMQ‐ApoE, IL‐23‐ApoE, and Card14‐ApoE, exposed to HFD for atherogenesis. Though they demonstrated that endothelial IL‐9R/STAT3 signaling directly promotes endothelial dysfunction and atherogenesis, the skin inflammation was still relatively acute (three 5‐day IMQ cycles), and the role of macrophage polarization was not systematically investigated. Dong et al. [31] performed single‐cell RNA‐seq (scRNA‐seq) on skin lesions from PSO patients with or without AS and identified CD11b^+^ monocyte‐derived macrophages as key players. They validated the GPIb‐CD11b axis in platelet‐macrophage crosstalk using an IMQ‐induced dermatitis model; however, the observed differences in plaque burden between groups were not pronounced. Zhu et al. [32] reported that adipose tissue macrophage–derived PPBP exacerbates PSO‐associated AS by inducing mitochondrial dysfunction in aortic endothelial cells. They used an IMQ‐ApoE model with IMQ applied 4 days/week for 4 weeks and performed PPBP knockout and neutralization studies. This model emphasizes the adipose‐vascular axis but did not systematically evaluate the reverse exacerbation [32].

The bidirectional exacerbation in PSO‐AS comorbidity points to a shared immunopathogenic axis. We propose that IL‐17A acts as a key upstream regulator of this vicious cycle [33]. Psoriatic skin inflammation is primarily driven by IL‐17–mediated pathways. Clinical evidence further supports the critical role of the IL‐17 axis in PSO, as anti‐PD‐1 therapy can induce PSO through IL‐17 overproduction [34]. Melanoma‐derived exosomes have been demonstrated to alleviate psoriatic inflammation by delivering miRNAs that directly target IL‐17A, thereby suppressing T‐cell activation [35]. IL‐17A significantly promotes tissue inflammation and upregulates the production of chemokines involved in pathogenesis, such as C‐C motif chemokine 20 (CCL20), C‐X‐C motif chemokine ligand 1 (CXCL1), and C‐X‐C motif chemokine ligand 8 (CXCL8) [36]. Additionally, IL‐17A receptors are widely expressed in the vascular wall. Binding of IL‐17A to its receptors induces the production of TNF‐α, IL‐1β, C‐C motif chemokine 2 (CCL2), and intercellular adhesion molecule 1 (ICAM‐1) [37]. The proatherosclerotic effect of IL‐17 is mainly mediated by these enhanced inflammatory responses. Studies in ApoE‐deficient mice have shown that inhibiting IL‐17A significantly reduces the atherosclerotic area, decreases maximal stenosis, and downregulates C‐C motif chemokine 5 (CCL5) expression, as well as levels of IL‐6, TNF‐α, and vascular cell adhesion molecule‐1 (VCAM‐1) [38]. Mechanistically, the sustained IL‐17A surge in comorbid mice may act upstream to promote M1 polarization, as IL‐17A is known to synergize with TNF‐α and IFN‐γ to lock macrophages into a proinflammatory phenotype [39, 40]. Previous studies confirmed the increase of CD68^+^ iNOS^+^ M1 macrophages and the decrease of CD163^+^ M2 macrophages in psoriatic lesions of the skin. Endosomal TLR7/9 activation by self‐nucleic acid‐antimicrobial peptide complexes, which are abundant in psoriatic skin, promotes M1 polarization. Conversely, molecules such as RGC‐32 and IL‐35 that support M2 differentiation are downregulated in psoriatic lesions [41]. In AS, macrophages adopt diverse phenotypes depending on the lesional microenvironment. M1‐like macrophages are driven by IFN‐γ, TLR ligands, and modified lipoproteins, producing IL‐1β, TNF‐α, and iNOS that promote plaque progression. Free cholesterol accumulation caused by defective efflux or scavenger receptor overactivity usually triggers ER stress, NLRP3 inflammasome activation, and mitochondrial oxidative stress, reinforcing M1 polarization and NC expansion [42]. Conversely, interventions that modulate the NF‐κB/NLRP3 pathway to promote M2 polarization or directly inhibit NLRP3‐mediated pyroptosis have been shown to alleviate inflammatory conditions and AS [43, 44]. Therefore, targeting M1 polarization may serve as a valuable target for PSO‐AS comorbidity. Use of TNF‐α inhibitors, a well‐known M1‐targeting therapy, not only ameliorated skin lesions but also significantly reduced MI risks [45]. The macrophage‐targeted nanomedicines also offer a promising translational insight for precisely modulating macrophage phenotypes in chronic inflammatory diseases like PSO‐AS [46].

In our results, the M1 polarization marker (CD86) was upregulated in the PSO‐AS aorta, compared with AS counterparts. Notably, proteomics and ML identified IFIT3 as a core M1‐associated protein in PSO‐AS skin, which was also upregulated in the aorta. IFIT3 belongs to the IFIT family, which has been characterized as highly selective markers of M1 macrophage polarization in proteomic studies of THP‐1 and primary human macrophages [47–49]. Subsequent bioinformatics analyses have identified IFIT3 as an immune‐related hub gene in AS, with high diagnostic value and positive correlations with memory B cells and resting dendritic cells [50, 51]. In the context of comorbidity, a network analysis of AS and COVID‐19 revealed that IFIT3 is a target of the transcription factor SPI1 and is upregulated in SARS‐CoV‐2–infected cardiomyocytes, linking IFIT3 to inflammatory responses in cardiovascular complications [52]. Furthermore, circulating classical monocytes from subjects with high lipoprotein(a), a causal risk factor for atherosclerotic cardiovascular disease (ASCVD), exhibited significantly increased IFIT3 gene expression. This phenomenon was not accompanied by changes in global chromatin accessibility, suggesting that IFIT3 upregulation may occur through transcription factor activity rather than epigenetic remodeling [53]. Given the high expression of IFIT3 in both skin and aorta of the PSO‐AS mouse, IFIT3 may serve as a key molecular link connecting psoriatic inflammation to AS progression, potentially by amplifying type I interferon responses and M1 polarization, thereby promoting proinflammatory cytokine secretion and plaque vulnerability. Macrophage‐specific Ifit3 deletion in the PSO‐AS mouse needed to define its role.

The advantages and limitations of our model are also obvious. Compared to genetically engineered models, our protocol relies on widely accessible chemical induction (IMQ) and dietary intervention (HFD), providing a cost‐effective platform for comorbidity research. Secondly, the model successfully replicates the bidirectional exacerbation observed in patients—where AS amplifies psoriatic skin inflammation, while PSO accelerates atherosclerotic plaque progression, leading to increased vulnerability. Thirdly, we pointed out that M1 progression may serve as the core mechanism in PSO‐AS comorbidity, and IFIT3 might be a promising biomarker of M1 polarization in PSO‐AS comorbidity.

However, several limitations are worth further consideration: First, some studies suggested that prolonged IMQ induction may induce local immune tolerance and potentially lead to systemic lupus erythematosus [54], potentially confounding mechanistic interpretations [55]. Lastly, clinical PSO‐AS comorbidities often exhibit temporal heterogeneity (e.g., PSO preceding AS by years). Our simultaneous induction protocol cannot dissect how disease order influences crosstalk—a critical gap for understanding early intervention strategies.

## 5. Conclusion

We successfully established a novel murine model of PSO‐AS comorbidity by integrating chronic IMQ–induced psoriatic inflammation with HFD‐driven AS in ApoE^−/−^ mice. This model robustly recapitulates the bidirectional exacerbation observed clinically and identifies M1 macrophage polarization as a central mechanism driving both skin and vessel pathology. This platform provides a critical tool for mechanistic exploration and therapeutic intervention in PSO‐AS comorbidity.

**Nomenclature**
AS: Atherosclerosis
ASCVD: Atherosclerotic cardiovascular disease
BC: Blank control
CCL2: C‐C motif chemokine 2
CCL5: C‐C motif chemokine 5
CCL20: C‐C motif chemokine 20
CD: Chow diet
CI: Confidence interval
CVD: Cardiovascular disease
CXCL1: C‐X‐C motif chemokine ligand 1
CXCL8: C‐X‐C motif chemokine ligand 8
CXCL10: C‐X‐C motif chemokine ligand 10
DEGs: Differentially expressed genes
DIA: Data‐independent acquisition
DT: Decision tree
FC: Fibrous cap
GO: Gene Ontology
GSEA: Gene set enrichment analysis
H&E: Hematoxylin and eosin
HFD: High‐fat diet
HR: Hazard ratio
ICAM1: Intercellular adhesion molecule 1
IE: Indirect effect
IFN‐γ: Interferon‐γ
IL‐23: Interleukin‐23
IL‐6: Interleukin‐6
IL‐17A: Interleukin‐17A
IL‐17F: Interleukin‐17F
IL‐1β: Interleukin‐1β
IF: Immunofluorescence
IMQ: Imiquimod
GM‐CSF: Granulocyte‐macrophage colony‐stimulating factor
KEGG: Kyoto Encyclopedia of Genes and Genomes
LASSO: Least absolute shrinkage and selection operator
LDL: Low‐density lipoprotein
MACEs: Major adverse cardiovascular events
MIs: Myocardial infarctions
ML: Machine learning
MS: Mass spectrometry
NC: Necrotic core
ORO: Oil red O
PASI: Psoriasis area and severity index
PCA: Principal component analysis
PSMs: Peptide‐spectrum matches
PSO: Psoriasis
qRT‐PCR: Quantitative real‐time PCR
RF: Random forest
RNA‐seq: RNA sequencing
scRNA‐seq: Single‐cell RNA sequencing
SVM: Support vector machine
TC: Total cholesterol
Tcfa: Thin‐cap fibroatheromas
Th‐17: T helper cell 17
TNF‐α: Tumor necrosis factor alpha
VCAM‐1: Vascular cell adhesion molecule‐1
VPI: Vulnerability plaque index.

## Author Contributions

Conceptualization: Fangshun Tan. Methodology: Fangshun Tan, Yu Jiang, Qi Pan, and Jiafei Li. Formal analysis: Yuting Xia and Jing Xu. Resources: Liang Zhao, Ziquan Hu, Wenjie Yan, and Jiajia Lan. Visualization: Menglu Liu, Mengyuan Wang, and Tianlang Zhao. Writing – original draft: Fangshun Tan and Ruizhi Wang. Supervision: Juan Tao and Weixian Yang. Funding acquisition: Jing Xu and Jiajia Lan.

## Funding

The work was supported by the grants from the National Natural Science Foundation of China (Grant 82573984), the Natural Science Foundation of Beijing, China (Grant 7264319), and the Postdoctoral Fellowship Program of China Postdoctoral Science Foundation (Grants GZC20251509 and 2025M772331).

## Ethics Statement

The authors have nothing to report.

## Consent

The authors have nothing to report.

## Conflicts of Interest

The authors declare no conflicts of interest.

## Supporting Information

Additional supporting information can be found online in the Supporting Information section.

## Supporting information


**Supporting Information 1** Figure S1: Representative images of the subitem and total PASI score of mouse dorsal skin. Representative dorsal skin images of mice showing different PASI subitem scores (erythema, scaling, and thickness; each 0–4) and corresponding total PASI scores (sum of three subitems, range 0–12). PASI scoring was performed independently by three investigators blinded to group allocation.

**Supporting Information 2** Figure S2: The animal experiment design in which ApoE−/− mice (6–8 weeks old) were subjected to PSO‐AS, AS, PSO, and BC groups over a 12‐week‐period. The PSO‐AS group was fed a HFD starting at week 0, followed by topical application of 50 mg 5% IMQ for 5 consecutive days, 2 days off, and repeated from week 8 until sacrifice at week 12. The AS group received HFD without IMQ and was sacrificed at week 12. The PSO group was fed a CD starting at week 0, with IMQ application following the same 5‐day on/2‐day off cycle from week 8 until sacrifice at week 12. The BC group received CD without IMQ and was sacrificed at week 12. To be mentioned, the 4‐week IMQ application in the PSO‐AS and PSO groups was separated into a 2‐week upper back IMQ application and a 2‐week lower back IMQ application. Arrows indicate the start of HFD/CD (black), initiation of IMQ treatment (red), and time of sacrifice (blue).

**Supporting Information 3** Figure S3: Machine learning feature selection process for identifying core differentially expressed proteins in PSO‐AS and PSO skin proteomics. (A) Boruta algorithm. Left: Boxplot of feature importance for shadow features and real features. Right: Importance history over iterations, showing how each feature’s importance evolves across random forest runs. Confirmed, tentative, and rejected features are distinguished. (B) LASSO regression. Left: Coefficient profile plot showing the shrinkage of coefficients as the penalty parameter λ increases. Each curve represents a gene. Right: 10‐fold cross‐validation error curve (binomial deviance) vs. log(λ). The left and right vertical dashed lines indicate λ.min (minimum deviance) and λ.1se (one standard error rule), respectively. Genes with nonzero coefficients at λ.min are selected. The optimal λ is 0.00494027. (C) DT algorithm. Bar plot of variable importance (overall score) for the top 8 genes identified by the decision tree. (D) SVM algorithm. Plot of root mean square error (RMSE) from cross‐validation versus the number of features. (E) RF algorithm. Left: Variable importance based on MeanDecreaseAccuracy. Right: Detailed ranking of the top genes with their MeanDecreaseGini values. (F) XGBoost algorithm. Feature importance bar plot. Importance values are normalized to sum to 1.

## Data Availability

Supporting data will be available upon reasonable request.
