# Supplementary material for: Macrophage‐Driven Bidirectional Exacerbation in Psoriasis‐Atherosclerosis Comorbidity: Insights From a Novel Mouse Model
Source: Mediators Inflamm. 2026 Jul 26;2026:1377824. doi: 10.1155/mi/1377824 (PMC13402896; doi:10.1155/mi/1377824)
Supplement: Supplementary file 1 — Supporting Information 1 Figure S1: Representative images of the subitem and total PASI score of mouse dorsal skin. Representative dorsal skin images of mice showing different PASI subitem scores (erythema, scaling, and thickness; each 0–4) and corresponding total PASI scores (sum of three subitems, range 0–12). PASI scoring was performed independently by three investigators blinded to group allocation. [file MI-2026-1377824-s001.pdf]

	PASI score (total)	thickness	erythema	scaling
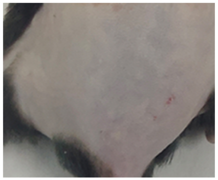	0	0	0	0
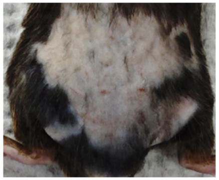	2-4	0-1	0-1	0-1
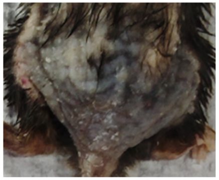	5-7	1-2	2-3	1-2
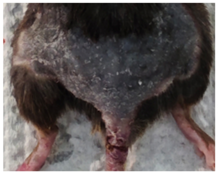	8-10	2-3	3-4	2-3
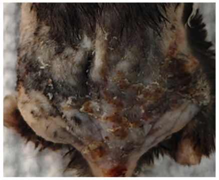	10-12	3-4	3-4	3-4
