# Supplementary figures and images for: Macrophage‐Driven Bidirectional Exacerbation in Psoriasis‐Atherosclerosis Comorbidity: Insights From a Novel Mouse Model
Source: Mediators Inflamm. 2026 Jul 26;2026:1377824. doi: 10.1155/mi/1377824 (PMC13402896; doi:10.1155/mi/1377824)

**A**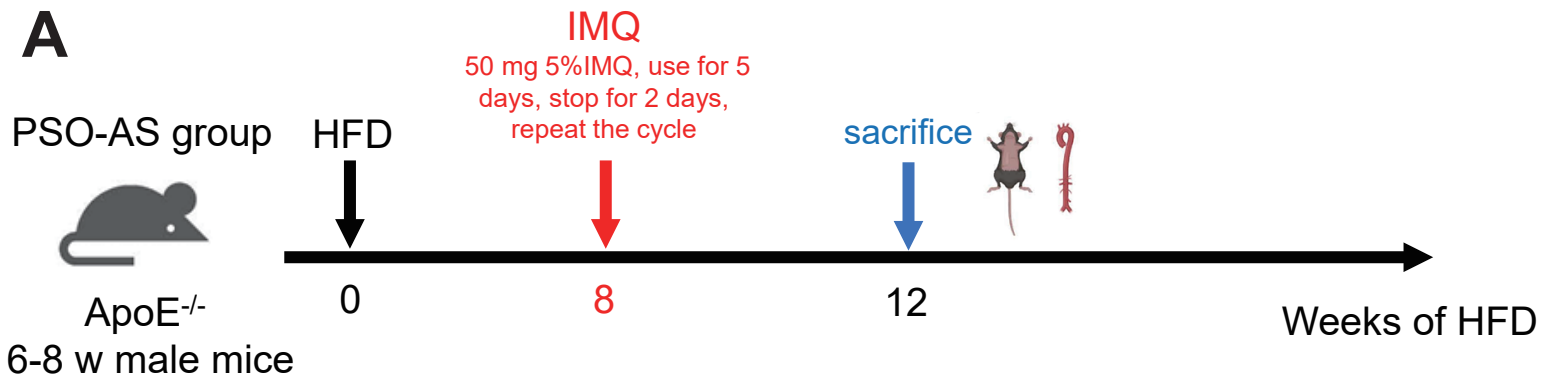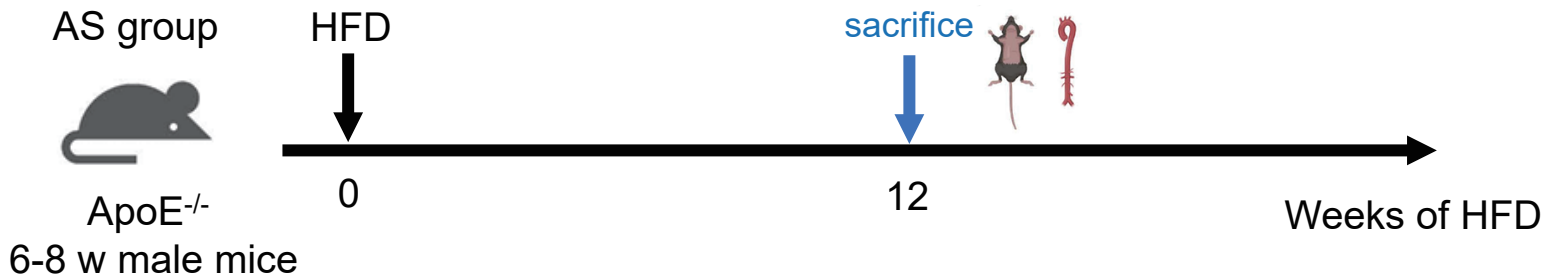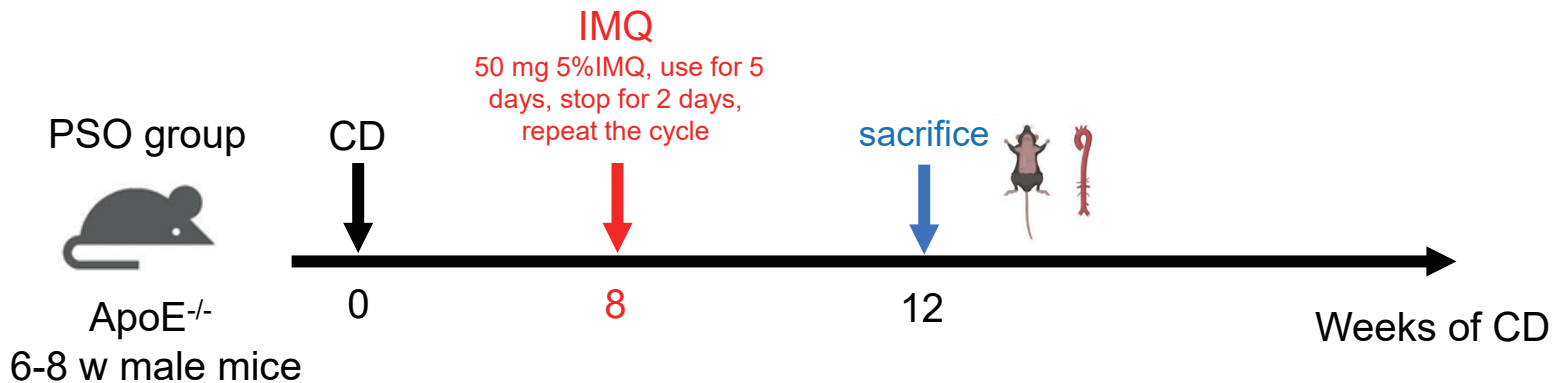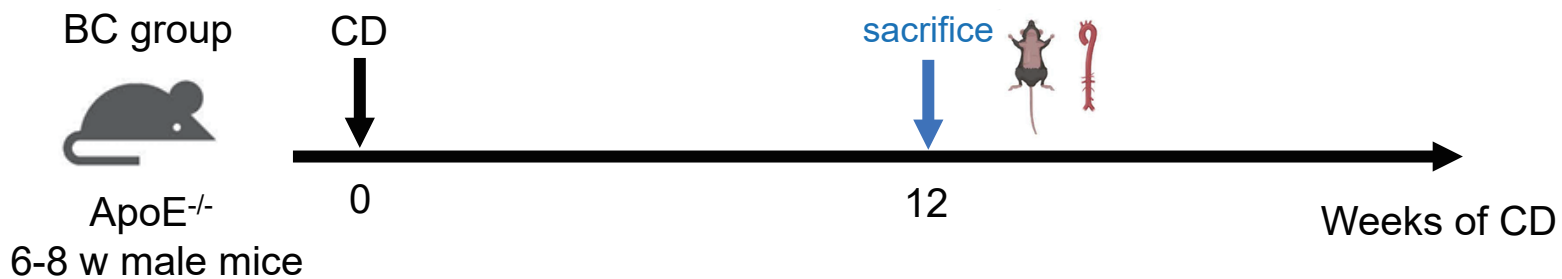

Supplement: Supplementary file 2 — Supporting Information 2 Figure S2: The animal experiment design in which ApoE−/− mice (6–8 weeks old) were subjected to PSO‐AS, AS, PSO, and BC groups over a 12‐week‐period. The PSO‐AS group was fed a HFD starting at week 0, followed by topical application of 50 mg 5% IMQ for 5 consecutive days, 2 days off, and repeated from week 8 until sacrifice at week 12. The AS group received HFD without IMQ and was sacrificed at week 12. The PSO group was fed a CD starting at week 0, with IMQ application following the same 5‐day on/2‐day off cycle from week 8 until sacrifice at week 12. The BC group received CD without IMQ and was sacrificed at week 12. To be mentioned, the 4‐week IMQ application in the PSO‐AS and PSO groups was separated into a 2‐week upper back IMQ application and a 2‐week lower back IMQ application. Arrows indicate the start of HFD/CD (black), initiation of IMQ treatment (red), and time of sacrifice (blue). [file MI-2026-1377824-s002.pdf]

**A**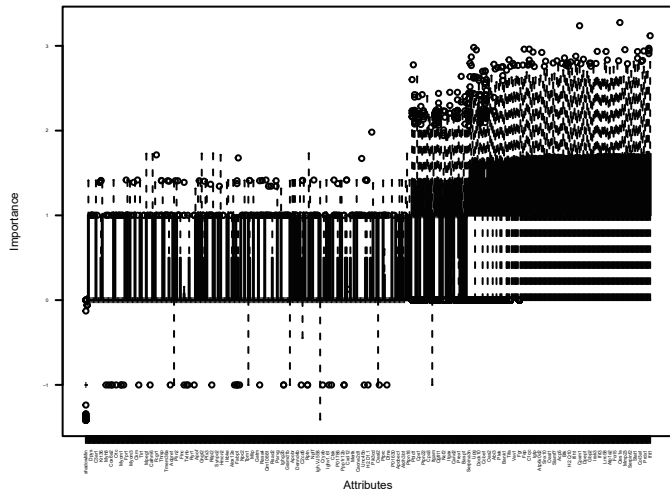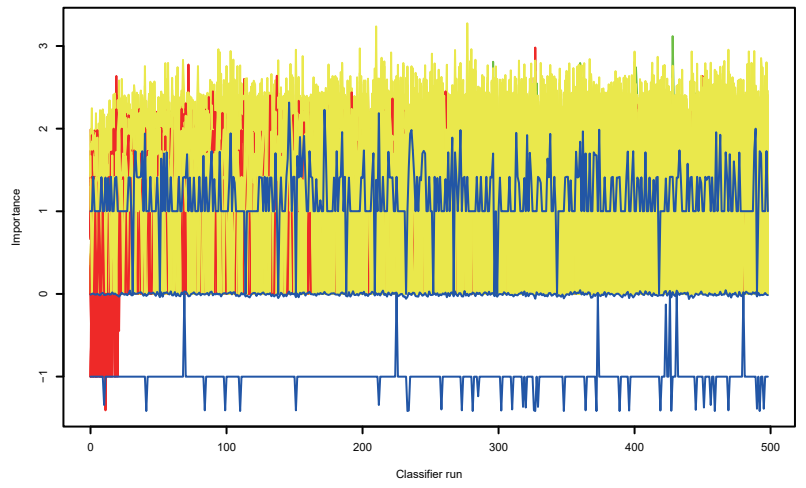**B**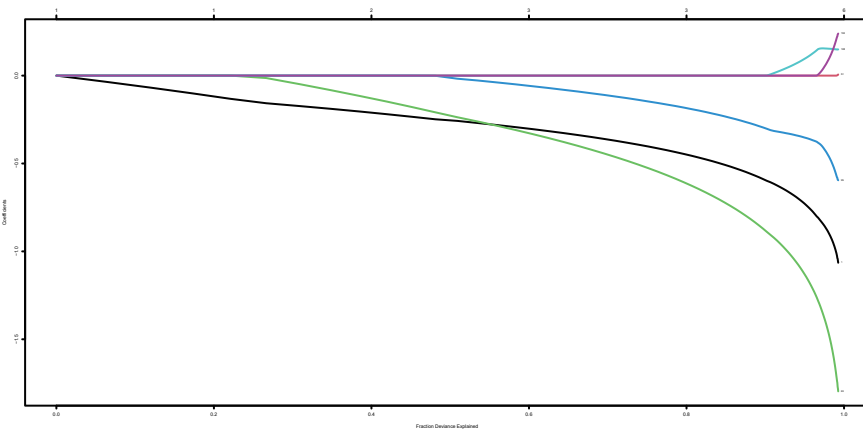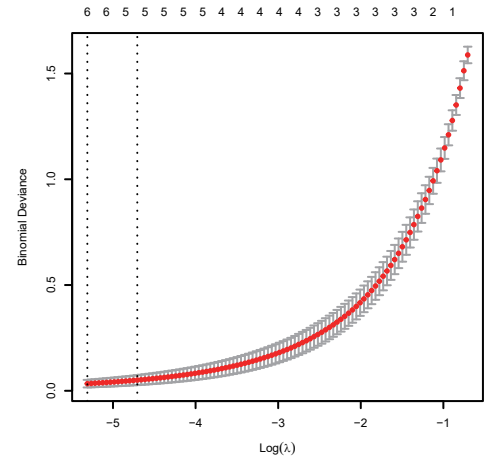**C**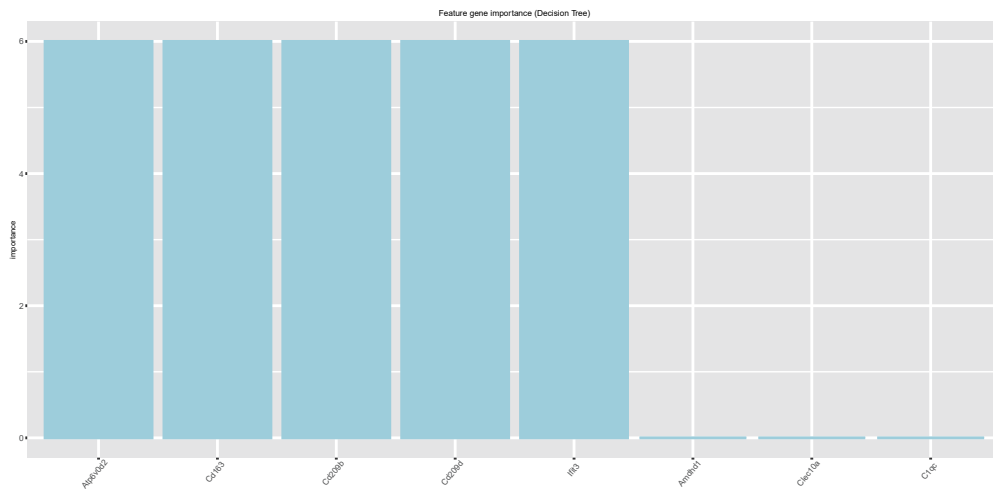**D**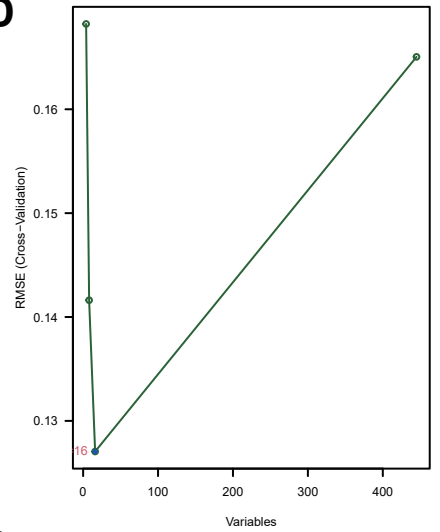**E**

Random forest

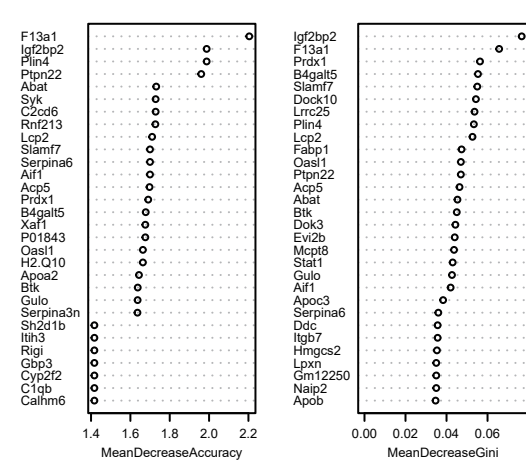

Random forest

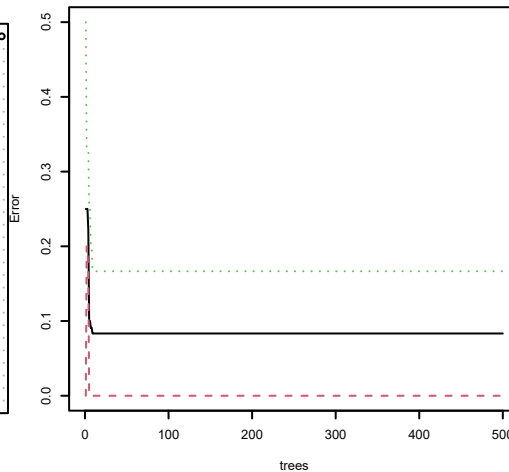**F**

Feature importance

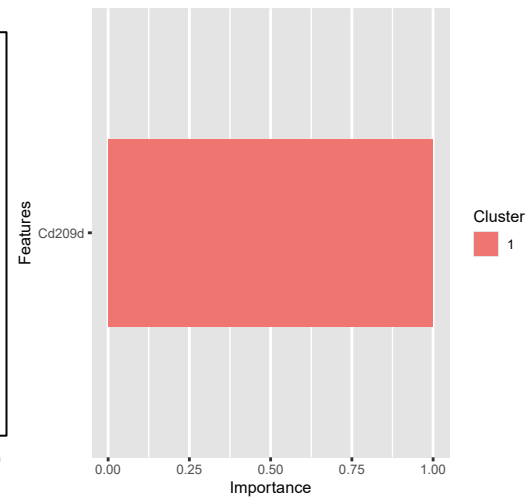

Supplement: Supplementary file 3 — Supporting Information 3 Figure S3: Machine learning feature selection process for identifying core differentially expressed proteins in PSO‐AS and PSO skin proteomics. (A) Boruta algorithm. Left: Boxplot of feature importance for shadow features and real features. Right: Importance history over iterations, showing how each feature’s importance evolves across random forest runs. Confirmed, tentative, and rejected features are distinguished. (B) LASSO regression. Left: Coefficient profile plot showing the shrinkage of coefficients as the penalty parameter λ increases. Each curve represents a gene. Right: 10‐fold cross‐validation error curve (binomial deviance) vs. log(λ). The left and right vertical dashed lines indicate λ.min (minimum deviance) and λ.1se (one standard error rule), respectively. Genes with nonzero coefficients at λ.min are selected. The optimal λ is 0.00494027. (C) DT algorithm. Bar plot of variable importance (overall score) for the top 8 genes identified by the decision tree. (D) SVM algorithm. Plot of root mean square error (RMSE) from cross‐validation versus the number of features. (E) RF algorithm. Left: Variable importance based on MeanDecreaseAccuracy. Right: Detailed ranking of the top genes with their MeanDecreaseGini values. (F) XGBoost algorithm. Feature importance bar plot. Importance values are normalized to sum to 1. [file MI-2026-1377824-s003.pdf]
